# Mechanism of medical hemorrhoid gel in relieving pruritus ani via inhibiting the activation of JAK2/STAT3 pathway

**DOI:** 10.3389/fmed.2024.1487531

**Published:** 2024-11-13

**Authors:** Zhongzhu Ai, Dongfeng Yuan, Jingyi Cai, Ruotong Dong, Wei Liu, Daonian Zhou

**Affiliations:** ^1^School of Pharmacy, Hubei University of Chinese Medicine, Wuhan, China; ^2^Hubei Shizhen Laboratory, Wuhan, China; ^3^Mayinglong Pharmaceutical Group Co., Ltd, Wuhan, China

**Keywords:** pruritus, hemorrhoid, medical hemorrhoid gel, JAK2/STAT3, skin-brain axis

## Abstract

**Background:**

Pruritus ani (PA), a neurofunctional dermatosis, is one of the most common complications of hemorrhoids, which seriously affects the quality of life of patients. Medical hemorrhoid gel (MHG), a product mainly composed of herbal medicine, is widely used for treatment of PA clinically. This study aim to assess the alleviating effect and mechanism of MHG on PA based on rectal epidermis-spinal cord-brain axis using animal models.

**Methods:**

A chloroquine-induced mouse itching model and a croton oil preparation-induced rat hemorrhoid model were established to evaluate anti-PA effect of MHG. Scratching behaviors of mice were recorded, and histopathology of mice skin and rat ano-rectal tissues was observed through H&E staining. Network pharmacology and western blotting were employed to explore potential mechanism of MHG.

**Results:**

The study indicated that MHG significantly alleviated chloroquine-induced skin itching and improved pathological injuries in mice skin and rat ano-rectal tissues. Network pharmacology suggested that MHG might regulate the JAK/STAT signaling pathway. Experimental findings showed that MHG significantly downregulated TRPV1 and TRPA1 in rectal tissue, c-Fos and GRPR in spinal cord tissue, and 5-HT1a protein in brain tissue, while upregulating TRPM8 protein in rectal tissue. Furthermore, MHG inhibited the activation of the JAK2/STAT3 signaling pathway in the rectal epidermis-spinal cord-brain axis.

**Conclusion:**

MHG improves PA by inhibiting the transmission of itching signals in rectal epidermis-spinal cord-brain axis via the JAK2/STAT3 signaling pathway, providing experimental evidence for its clinical application.

## 1 Introduction

Hemorrhoids are the most common anal and rectal disease, leading to symptoms such as bleeding, prolapse, and itching. Factors like prolonged straining, irregular bowel habits, and genetic predisposition contribute to its prevalence, making it the most widespread colorectal disorder globally (1). The global prevalence of hemorrhoids is as high as 11%, and pruritus ani (PA) induced by hemorrhoids is a localized itch occurring in the anal or genital area. It is classified as a neurofunctional dermatosis, presenting symptoms like skin redness, erosion, swelling, and intense itching, severely impacting sleep and even mental health (2, 3). The prevailing medical consensus is that PA arises when the skin around the anus is stimulated by factors such as feces, chemicals, or physical irritation, leading to the release of chemical mediators like histamine and kinins. These mediators activate the itch receptors located in the nerve endings of the epidermis and superficial dermis of the anus, triggering the sensation of itching (4). However, the modern understanding of mechanism of PA remains limited, particularly the itch signal transmission from the anal epidermis to the brain remains unclear. Unfortunately, there is currently no fully satisfactory treatment for PA. PA is persistent and prone to recurrence, significantly disrupting daily lives and work of patients (5). Therefore, understanding the mechanism of itch signal transmission in PA is crucial for developing new medications.

As is known, itching in hemorrhoids occurs when skin receptors detect stimuli, sending impulses through sensory nerve fibers to the dorsal root ganglia (DRG) and trigeminal ganglion (TG). These impulses are then transmitted via the spinal dorsal horn (SDH) to the cerebral cortex (6), highlighting the crucial role of the skin-spinal cord-brain axis in processing itch signals. The gastrin-releasing peptide receptor (GRPR) expressed in SDH neurons induces itching, with the GRP/GRPR axis mediated by the 5-HT1a receptor being a key pathway in regulating itch perception (7, 8).

Traditional Chinese medicine, whether applied externally or taken internally, can effectively alleviate symptoms in patients with hemorrhoids, particularly in reducing pain and itching and improving quality of life (9). Medical hemorrhoid gel (MHG) is commonly used in clinical practice for treating hemorrhoids, with observations showing significant improvements in hemorrhoid symptoms, such as hemostasis and pruritus. It is primarily composed of *Hamamelis mollis* Oliv. (Jinlvmei), *Cinnamomum camphora* (L.) Presl (Bingpian), and recombinant collagen. Studies have indicated that Jinlvmei can alleviate symptoms such as bleeding, swelling, pain, and itching, making it particularly effective in the early stages of hemorrhoid treatment (10). Bingpian possesses analgesic, anti-inflammatory, and antioxidant properties. It has been shown to relieve both acute and chronic itching in mice by activating TRPM8 in sensory neurons and inhibiting TRPA1 (11, 12). Recombinant collagen reduces inflammation, promotes collagen deposition and vascular regeneration, and demonstrates strong anti-inflammatory and regenerative effects (13). Additionally, its excellent biocompatibility enhances the bioavailability and therapeutic efficacy of compatible drugs (14). Despite its widespread use in China for treating hemorrhoids, the exact mechanism by which MHG relieves itching remains unclear.

Based on the above reports, this study investigates the rectum epidermis-spinal cord-brain axis involved in itch signal transmission. By establishing *in vitro* animal models, it evaluates the anti-itch effects of MHG and elucidates the molecular mechanisms through which MHG alleviates PA. This research aims to provide experimental evidence supporting the clinical application of MHG in treating hemorrhoidal pruritus.

## 2 Materials and methods

### 2.1 Drug preparation and reagents sources

Medical Hemorrhoid Gel (20230103) is provided by Hunan Renxin Biotechnology Co., Ltd. (Changsha, China). This product utilizes 50% ethanol extract of *Hamamelis mollis* Oliv, which has been tested and qualified by HPLC. It is prepared according to the ratio of 50:40:5 of *Hamamelis mollis* Oliv. extract, bingpian, and recombinant collagen, mixed with excipients, and manufactured through standardized preparation processes and quality control.

Titanoreine (20,230,103, Xi’an Yangsen Pharmaceutical Co., Ltd., China). Croton oil (C13225402) and chloroquine (W15J12S137893) were both purchased from Shanghai Macklin Biochemical Technology Co., Ltd. (Shanghai, China). Pyridine (P11511) was purchased from Shanghai Aladdin Biochemical Technology Co., Ltd. (Shanghai, China). Phosphatase inhibitor (MB12707) were provided by Meilunbio (Dalian, China).

### 2.2 Animals

Forty male SPF-grade C57BL/6 J mice weighing 20–22 g and twelve male SPF-grade SD rats weighing 200–220 g were provided by Henan Skebes Biotechnology Co., Ltd. (Anyang, China) with license numbers SYXK (E) 2017-0067 and SCXK (Yu) 2020-0005. Throughout the experiment, the animals were kept in regular experimental conditions at the Animal Experimental Center of Hubei University of Chinese Medicine, having unlimited accessibility to water and food. All experimental in this study were according to standards set by the Animal Experiment Ethics Committee of Hubei University of Chinese Medicine and in accordance with the ARRIVE guidelines. All experimental protocols were approved by the Animal Experiment Ethics Committee of Hubei University of Chinese Medicine on November 1, 2023, and the experiments were conducted from November 4, 2023, to December 30, 2023 (Permit No.: SYXK2023-0067-ZYZYZX2023-042).

### 2.3 Grouping and intervention

After acclimatization for 3 days, forty mice were split into four group at random: Control, Chloroquine, Chloroquine +MHG (5.0 g/kg), and Chloroquine + Titanoreine (5.0 g/kg), with 10 mice in each group. Twelve rats were assigned into four group at random: Control, COP, COP+MHG (1.0 g/kg), and COP+ Titanoreine (1.0 g/kg) groups, with 3 rats in each group. In this study, the selection of the dosage for animal administration was based on the clinical dosages of MHG and Titanoreine, which was determined by converting the dose per unit weight of the human body to the weight of the animal and combining it with the results of our preliminary experiments.

As previously described, itching was induced by subcutaneous injection of chloroquine (15). Briefly, prior to the experiment, the mice were shaved of their neck and back hair. Aside from the Control group, 30 μL of 10 μg/μL chloroquine solution was subcutaneously injected into the neck and back of the remaining mice to establish the itching model. The mice in the control group received an injection of the same volume of physiological saline. After injection, the needle was kept in place for about 5 s to prevent leakage of the solution. Following modeling 5 min, MHG corresponding to body weight was applied with disposable gloves to the neck and back skin of mice in Chloroquine +MHG group. Titanoreine corresponding to body weight was applied with disposable gloves to the neck and back skin of mice in Chloroquine + Titanoreine group. Then, observe the scratch behavior of each mouse.

As described previously, hemorrhoids were induced using croton oil (16). Briefly, the COP was prepared by mixing distilled water, pyridine, ether, and a 6% croton oil ether solution in a ratio of 1:4:5:10. Except for Control group, all rats had 0.16 mL of COP injected into the rectum, and the injection was held for 5 s to prevent leakage. After 30 min of contact with COP on the rectal mucosa, MHG corresponding to body weight were immediately administered the rectum 2 cm from the anal verge using an enema tube in COP+MHG group. Titanoreine corresponding to body weight were immediately administered the rectum 2 cm from the anal verge using an enema tube in COP+ Titanoreine group. Then, observe the swelling of the anus in each rat.

### 2.4 Sample collection

After 30 min of drug treatment, mice were euthanized by cervical dislocation. The skin from the sensitized area of the back of each group of mice and the same area of skin from the control group mice were excised and fixed overnight in 4% paraformaldehyde solution.

After 8 h of drug treatment, rats were deeply anesthetized by intraperitoneal injection of 2% pentobarbital sodium at a dose of 0.4 mL/100 g. Subsequently, the rats were fixed on the operating table, and the hair around the anal area was shaved off. The skin along the abdominal midline was incised to expose the rectum. Using fine surgical instruments, the rectal tissue was carefully separated and 2 cm of ano-rectal tissue was excised from the anus. The skin along the dorsal midline was also incised to expose the vertebral canal where the spinal cord is located. The vertebral canal from segments C3 to C7 was excised, and a syringe filled with physiological saline was used to carefully flush out and separate the spinal cord tissue from the vertebral canal. The skin of the head was cut open with tissue scissors to expose the skull, and the cranial cavity was opened. The cerebral hemispheres were separated using tissue forceps. Except for a portion of the rectal tissue used for fixation, all other tissues were rapidly frozen in liquid nitrogen after removal and stored at-80°C for subsequent research.

### 2.5 Scratching behavior recording

Mice from each group were placed in observation cages similar to their housing environment, with HYS-001 high-definition camera (HYUNDAI, Korea) positioned overhead to record their scratching behavior for 30 min. A method for evaluating scratching frequency was as follows: a complete scratching action was considered when a mice raised its back paw off the bottom of the cage and scratched its skin until the back paw went back to the ground or was licked, counting as one scratching event (17). Two experimenters independently recorded and evaluated the scratching frequency of the mice.

### 2.6 Histopathology analysis

After fixation for 24 h, fresh mouse skin tissue and rat rectal tissue were dehydrated, embedded in paraffin, and sectioned into 4 mm slices using an automatic rotary microtome (Leica, Germany). The slices were deparaffinized and stained with H&E staining kit (S191003, Pinofly, China) to observe the histological features of tissue damage in mouse skin and rat rectum.

### 2.7 Network pharmacology procedures

In our previous research, we combined UPLC-QTOF-MS/MS and GC–MS/MS to analyze the main chemical components of MHG. By comparing the ion fragments with the literature (18–20), we identified 20 compounds. The total ion flow chromatogram and information on the compounds is presented in the Supplementary material.

Twenty compounds identified from MHG were collected in SDF format from the PubChem database.^1^ The SMILES of compounds were gathered and inputted into the SwissTargetPrediction^2^ to collect predicted drug targets. Then, associated targets of pruritus were collected, respectively, from the GeneCards database,^3^ OMIM database,^4^ and DisGeNet database.^5^ All collected targets were merged and duplicates removed to obtain disease-related targets. The drug and disease targets were imported into Veeny 2.1.0^6^ to generate a Venn diagram, obtaining the intersection targets of MHG for pruritus. Cytoscape 3.10.1 was used to build a compound-target-disease network. Following importation of the effective targets to STRING database,^7^ the species *Homo sapiens* was chosen to obtain the data, and then, Cytoscape 3.10.1 software was utilized to construct the Protein–Protein Interaction (PPI) network. The intersection targets were uploaded into the DAVID database^8^ for Gene Ontology (GO) and Kyoto Encyclopedia of Genes and Genomes (KEGG) enrichment analysis. GO function and KEGG pathway analyses selected entries with *p* ≤ 0.01. The bioinformatics platform^9^ was utilized to visualize the top-ranked gene functions and KEGG pathways.

### 2.8 Western blotting

Appropriate amounts of ano-rectal tissue were homogenized to extract total protein, and the BCA protein assay kit (Gbcbio, China) was used to measure the protein content. After being denatured for ten to fifteen minutes in boiling water, the extracted protein was cooled to room temperature and then kept at-20°C. Gel electrophoresis was performed to separate the proteins using SDS-PAGE, and target bands were cut according to markers, washed, and transferred onto PVDF membranes (IPVH00010, Millipore, USA). After blocking for two hours in TBST solution containing 5% skim milk, the PVDF membrane was incubated with primary antibodies for a whole night at 4°C. After carefully washing the PVDF membrane five times with TBST, it was incubated for two hours with a species-specific antibody conjugated with HRP (1:10000). Excess secondary antibody was removed by washing with TBST, and the ECL reagent (KF8003, Affinity Bioscience, USA) mixed with stable peroxidase solution was dripped onto the PVDF membrane for visualization. Image J software was employed to examine the grayscale values, with *β*-actin used as an internal reference. Primary antibody information is shown in Table 1.

**Table 1:** The information and dilutions of primary antibody.

Antibody	Lot	Producer	Dilutions
TRPA1	A8568	Abclonal (Wuhan, China)	1:500
β-actin	66,009-1-Ig	Sanying (Wuhan, China)	1:30000
IL-6	BS-0782R	Bioss (Beijing, China)	1:1000
5-HT1a	BS-1124R	Bioss (Beijing, China)	1:1000
STAT3	9,139	CST (USA)	1:1000
p-STAT3	9,145	CST (USA)	1:2000
JAK2	AF6022	Affinity Bioscience (USA)	1:1000
p-JAK2	AF3024	Affinity Bioscience (USA)	1:1000
TRPV1	DF10320	Affinity Bioscience (USA)	1:1000
TRPM8	DF7966	Affinity Bioscience (USA)	1:2000
GRPR	DF2815	Affinity Bioscience (USA)	1:1000
c-Fos	AF0132	Affinity Bioscience (USA)	1:2000

### 2.9 Statistical method

The mean ± standard deviation (SD) was used to express all of the obtained data. Statistics analysis and data visualization were performed using OriginPro 2021 software. For comparisons between different groups, one-way analysis of variance (ANOVA) was employed. The statistical differences between the groups were assessed using the Tukey test, with *p* < 0.05 being considered statistically significant.

## 3 Results

### 3.1 MHG reduced the tendency to scratch of mice induced by chloroquine

**Figure 1:**
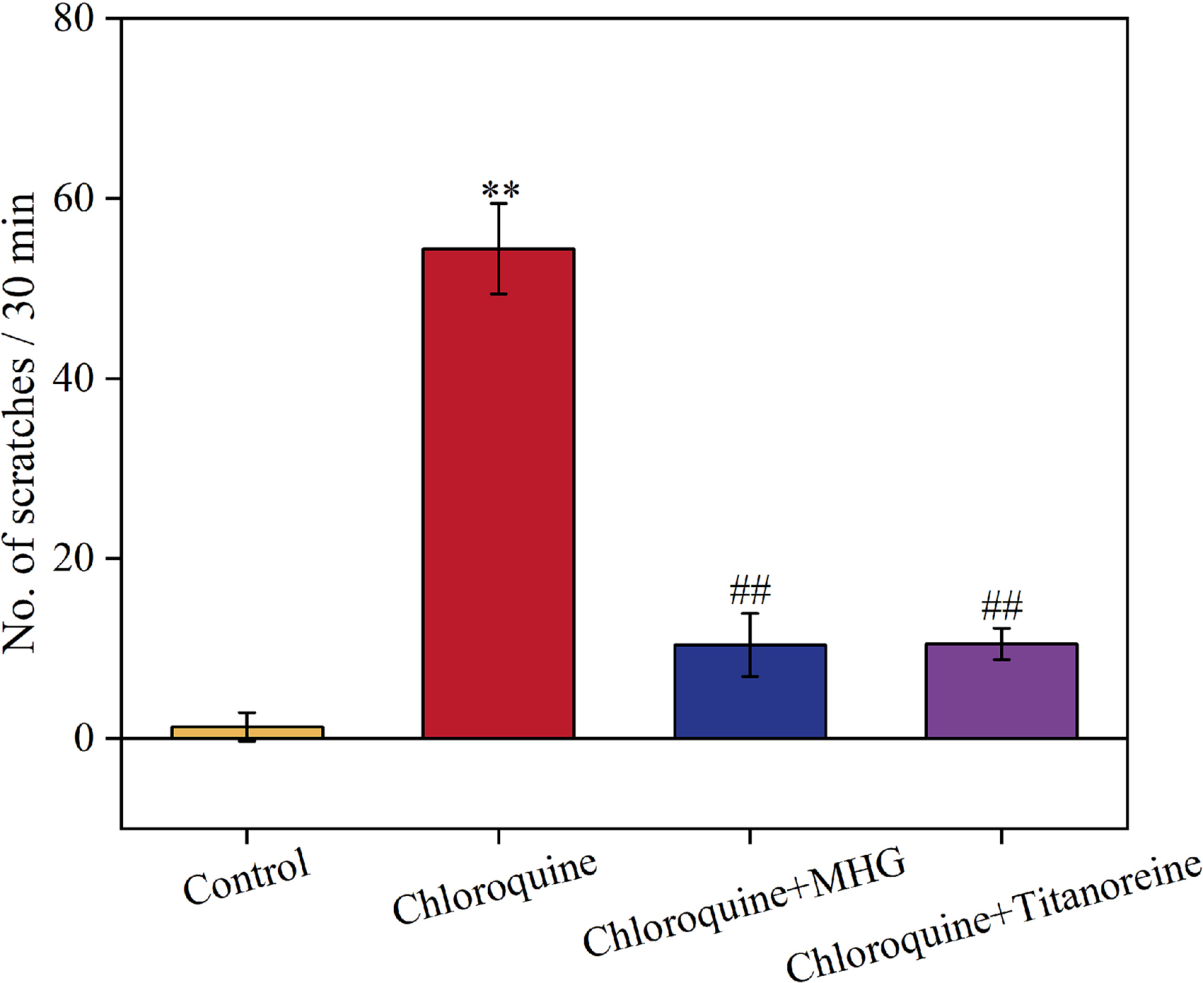
Scratching situation of mice within 30 min (*n* = 10). Data are presented as the mean ± SD, Compared with the control group; *p < 0.05, **p < 0.01, ***p < 0.001, compared with the COP group, #*p* < 0.05, ##*p* < 0.01, ###*p* < 0.001.

In this study, Titanoreine, a widely used anti-hemorrhoidal drug clinically known for its efficacy, safety, and rapid action in relieving of pain, pruritus, and inflammation, was selected as the positive control in this study (21, 22). The difference in scratching behavior before and after the experiment can be used to measure the antipruritic effect of the drug. The greater the difference in scratching behavior before and after administration, the more effective the drug is considered to be. The effective scratching frequencies within 30 min for each group of mice are shown in Figure 1. The scratching frequency of mice induced by chloroquine increased considerably within 30 min in comparison to the Control group (*p* < 0.01). Treatments with MHG and Titanoreine significantly reduced scratching frequency in comparison to the chloroquine group (*p* < 0.01). This suggests that MHG effectively alleviates scratching behavior induced by chloroquine in mice, with a comparable effect to Titanoreine.

### 3.2 MHG improves pathological damage to the skin of pruritus mice and ano-rectal tissues of hemorrhoid rats

**Figure 2:**
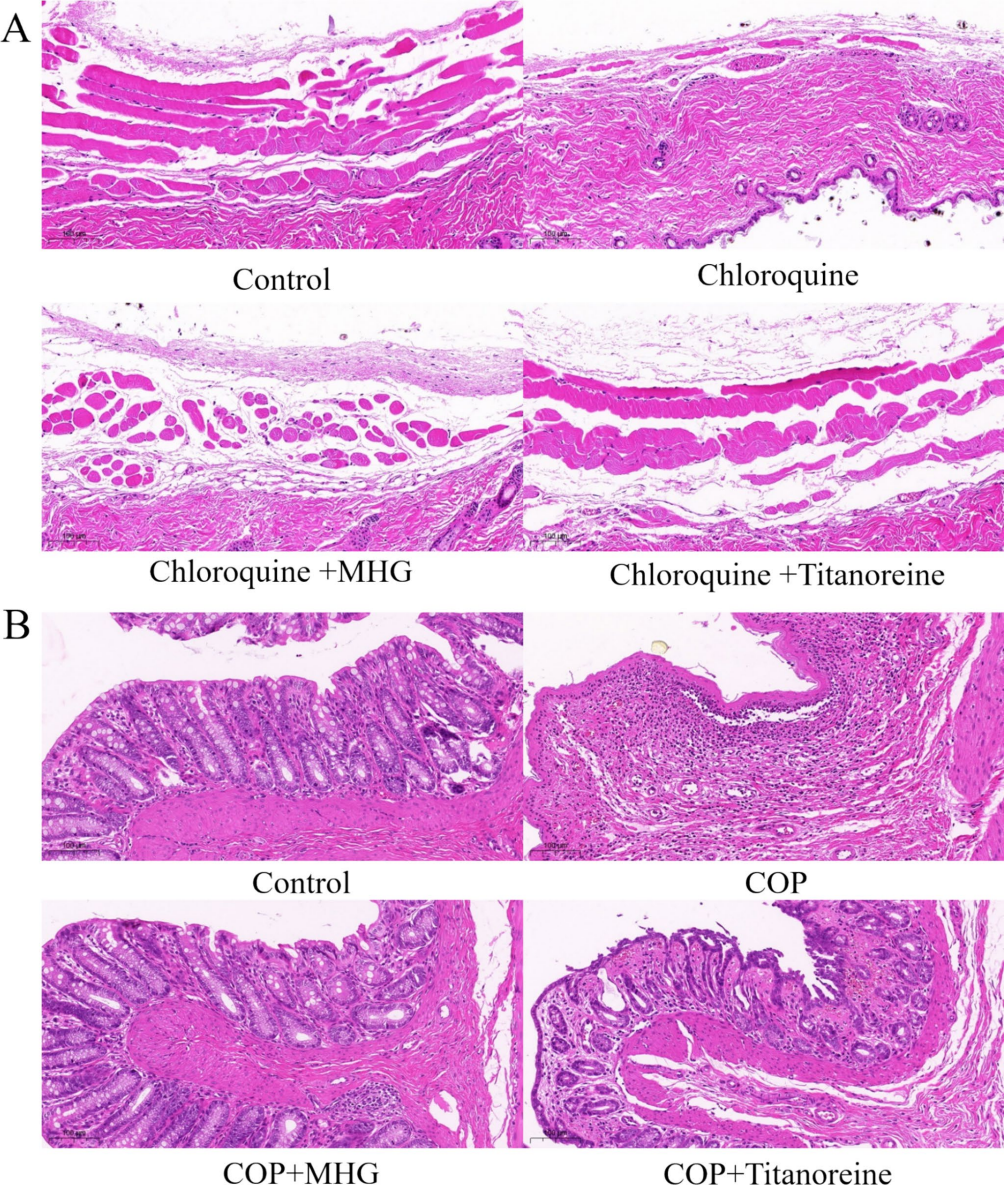
Histopathological staining analysis (HE staining ×100). (A) Pathological changes in skin tissue of the mice. (B) Pathological changes in the ano-rectal tissues of rats.

As shown in Figure 2A, the histopathological observations of the dorsal skin tissues of mouse showed that epidermal and dermal structures of skin were intact, with normal morphology and orderly cell arrangement, and no abnormal changes such as edema were observed in the Control group. Whereas the dermis was swollen and deformed, the squamous epithelium was shed, and inflammatory cell infiltration was evident in chloroquine-induced mouse, exhibiting severe histopathological damage. By contrast, there was some improvement in the pathological morphology of skin tissues after treatment with MHG and Titanoreine, characterized by widened dermal spaces, reduced shedding of squamous epithelium, and decreased inflammatory cell infiltration.

As shown in Figure 2B, the histopathological observations of the ano-rectal tissues of rats shown that the ano-rectal tissues in the Control group exhibited normal tissue structure, with intact villus structures arranged neatly. While the ano-rectal tissues of COP-induced rats showed significant acute inflammatory responses, accompanied by severe edema, disrupted villus structures, and infiltration of inflammatory cells, severe mucosal layer necrosis, vascular dilation, and local congestion bleeding. Instead still, the pathological damage to the ano-rectal tissues of rats was significantly reduced after treatment with MHG and Titanoreine, characterized by restored villus structures, reduced tissue edema, and decreased infiltration of inflammatory cells.

### 3.3 Network pharmacology predicts the potential mechanisms of MHG action

**Figure 3:**
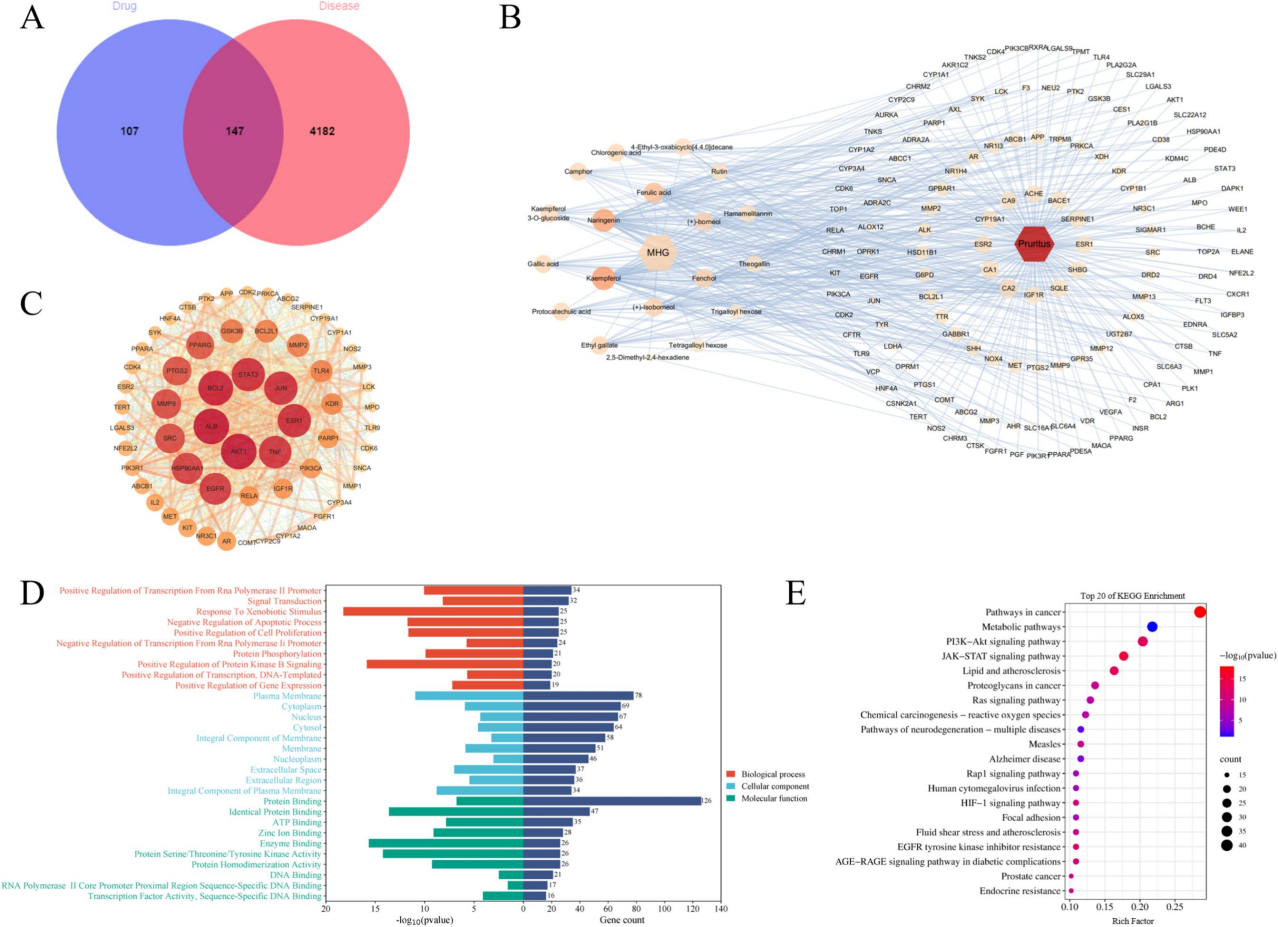
The mechanism of action of MHG is related to the JAK/STAT signaling pathway. (A) Veen diagram of compounds and disease targets. (B) Compounds-Targets-Disease network, the greater the degree value of the node, the higher the importance in the network. (C) PPI network. The larger the node, the greater the degree value of the node, the higher the importance in the network. (D) GO annotation analysis. (E) KEGG pathway enrichment analysis.

Since this product is a topical formulation, and after the drug dissolves in the rectal cavity, it is absorbed by the capillaries or lymphatic vessels in the submucosa of the rectum. Most of the drug enters the systemic circulation directly through the inferior rectal vein, avoiding the first-pass effect of the liver (23). Considering that recombinant collagen has a large molecular weight and is not easily absorbed (24), pharmacokinetic parameters such as oral bioavailability are not set for screening chemical components. Instead, 20 compounds obtained from preliminary analysis by HPLC–MS and GC–MS are selected for further analysis. Excluding one compound (gallocatechin) from MHG without corresponding target genes, the remaining 19 compounds were predicted for target genes using the SwissTargetPrediction database. An overall of 254 compound targets were found after deleting duplication target genes. Additionally, after removing duplicate target genes, 4,329 disease targets associated with pruritus were identified from three databases. Taking the intersection of compound and disease targets, 147 mapped targets were obtained, as shown in Figure 3A. To further highlight the correlation between drug components and main targets, Cytoscape 3.10.1 was used to import the 147 intersection targets of compounds and diseases to construct the C-T-D network, as shown in Figure 3B. Among them, the higher the degree value of a compound represents its more important role in the network. The top five compounds according to degree are Kaempferol, Naringenin, Ferulic acid, Fenchol, (+)-Isoborneol, and (+)-Borneol, mainly involving targets such as CA2, NR1H4, TRPM8, GRP35 and so on. Obviously, one compound can modulate multiple targets, and the same target may be modulated by multiple compounds, which indicates that MHG components have a synergistic regulatory effect in the treatment of itch.

To further clarify the core targets, sixty targets with Degree value higher than the median of 26 were chosen to construct the PPI network, as shown in Figure 3 (C). Clearly, some genes such as AKT1, ALB, BCL2, STAT3, JUN, ESR1, and TNF hold important positions within the network, indicating their strong relevance to the pathogenesis of pruritus and their potential as key targets for drug intervention. In addition, to illustrate potential target roles, functions, and signaling pathways, the DAVID database was used for gene annotation and visualization. GO functional enrichment analysis indicates that a total of 845 entries were obtained, with 609 entries for biological processes (BP), 81 entries for cellular components (CC), and 155 entries for molecular functions (MF). Figure 3D displays the top 10 entries in BP, CC, and MF. The main GO functional items involved include Signal Transduction, Cytoplasm, Protein Binding and so on. KEGG enrichment analysis can predict the roles of protein target interaction networks in various cellular activities and identify key protein targets and their associated pathways. 151 major pathways in all were collected from the KEGG enrichment analysis and Figure 3E displays the top 20 pathways of these pathways. These findings indicate the pathways associated with key targets primarily involve Pathways in cancer, Metabolic pathway, P13K–Akt signal pathway, JAK–STAT signaling pathway, Lipid and atherosclerosis and so on. These pathways play a vital role in immune response, cancer, inflammation, metabolism, measles, and psychiatric disorders. Based on these data and the combination of key targets, the JAK–STAT signaling pathway might be crucial, which will be verified in subsequent experiments.

### 3.4 MHG inhibits the transmission of PA signals via the rectum epidermis-spinal cord-brain axis

**Figure 4:**
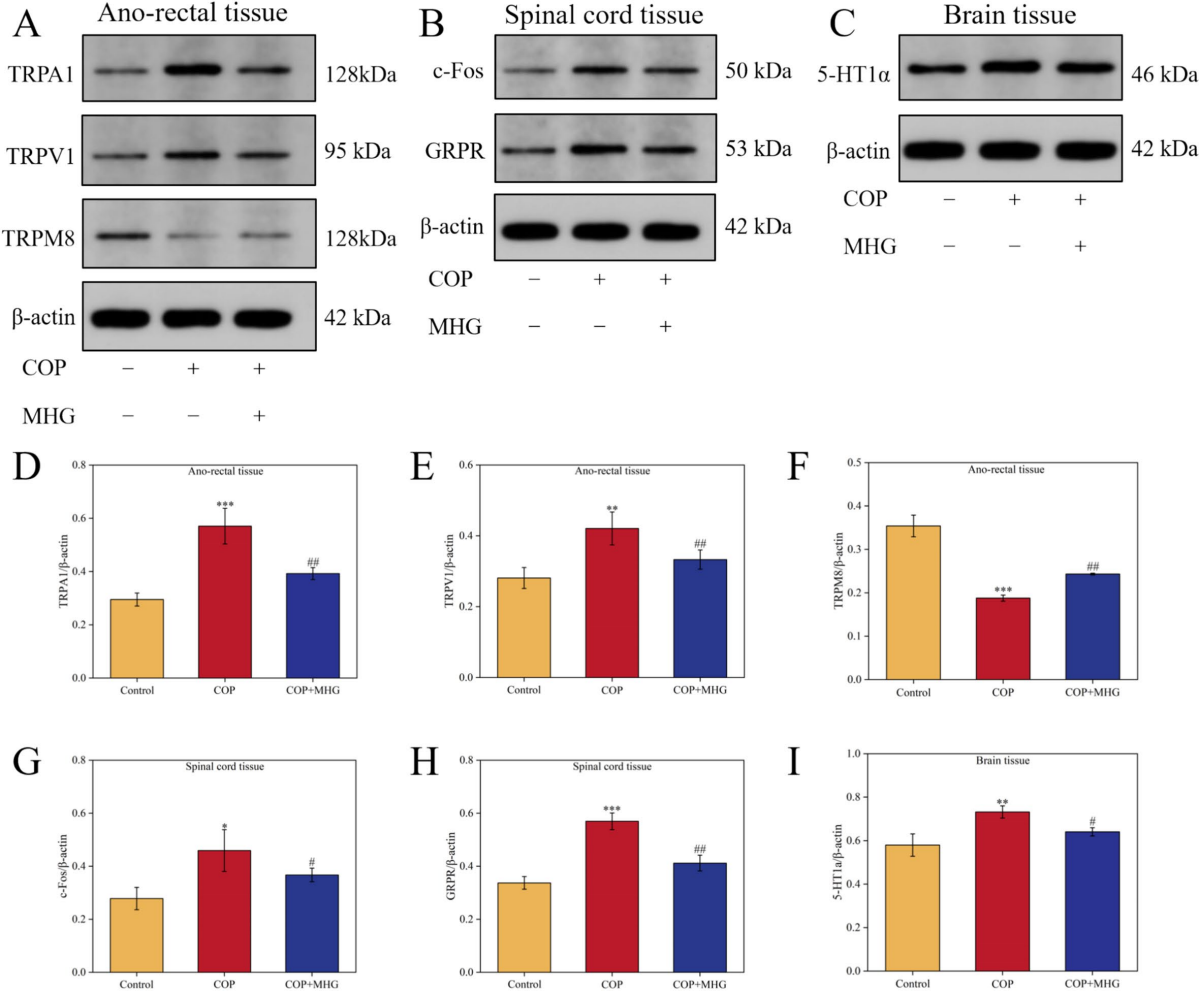
The effect of MHG on the expression of rectal-spinal-brain axis related proteins. (A) The representative bands of TRPA1, TRPV1 and TRPM8 were determined by Western blotting. (B) The representative bands of c-Fos and GRPR were determined by Western blotting. (C) The representative bands of 5-HT1a were determined by Western blotting. Protein relative expression level of TRPA1 (D), TRPV1 (E), TRPM8 (F), c-Fos (G), GRPR (H), 5-HT1a (I) (*n* = 3). Data are presented as the mean ± SD, Compared with the control group; ^*^p < 0.05, ^**^p < 0.01, ^***^p < 0.001, Compared with the COP group, *^#^p* < 0.05, *^##^p* < 0.01*, ^###^p* < 0.001. Uncropped raw data can be found in Supplementary Figure S3.

TRPA1, TRPV1, and TRPM8 are three important Transient Receptor Potential (TRP) channels, widely distributed in epidermal tissues, and play critical roles as skin receptors in the transmission of itching signals (25). In this study, Western Blotting was used to detect the relative expression levels of TRPA1, TRPV1, and TRPM8 proteins in rat anal rectal tissues, as shown in Figure 4A. From Figures 4D–F, the relative expression levels of TRPA1 and TRPV1 proteins in the COP group were significantly increased, while the relative expression level of TRPM8 protein was significantly decreased in comparison with the control group (*p* < 0.01 or *p* < 0.001). In contrast, the relative expression levels of TRPA1 and TRPV1 proteins were markedly decreased, while the relative expression of TRPM8 protein was significantly increased after treatment with MHG (*p* < 0.01).

When the skin is stimulated by itching, the released GRP from sensory nerve terminals acts on the GRPR on neurons in the spinal dorsal horn, activating these neurons and thus transmitting itching signals. An increase in c-Fos expression is a marker of neuron activation (26). In this study, Western Blotting was used to detect the relative expression levels of c-Fos and GRPR proteins in rat spinal cord tissues, as shown in Figure 4B. From Figures 4G,H, when comparison to the Control group, the relative expression levels of c-Fos and GRPR proteins in the COP group were markedly elevated (*p* < 0.05, *p* < 0.001). After treated with MHG, the relative expression levels of c-Fos and CRPR proteins dramatically decreased when comparison to the COP group (*p* < 0.05, *p* < 0.01).

The 5-HT1a receptor is a subtype of serotonin (5-HT) receptor, widely distributed in multiple regions of the brain, and is considered a key protein in the brain for processing and perceiving itching signals (27). In this study, Western Blotting was used to detect the relative expression levels of 5-HT1a protein in rat brain tissues, as shown in Figure 4C. From Figure 4I, the relative expression level of 5-HT1a protein in the COP group was noticeably increased when comparison with the Control group (*p* < 0.01). By contrast, the relative expression levels of 5-HT1a protein in the hemorrhoid rat were significantly decreased after treatment with MHG (*p* < 0.05).

The above results indicate that MHG can inhibit the transmission of itching signals in the rectal epidermis-spinal cord-brain axis by regulating the relative expression levels of TRPA1, TRPV1, TRPM8 in the rectum, c-Fos, GRPR in the spinal cord, and 5-HT1a in the brain of hemorrhoid rats.

### 3.5 MHG inhibits the activation of the JAK2/STAT3 signaling pathway in the rectal epidermis-spinal cord-brain axis of hemorrhoid rats

**Figure 5:**
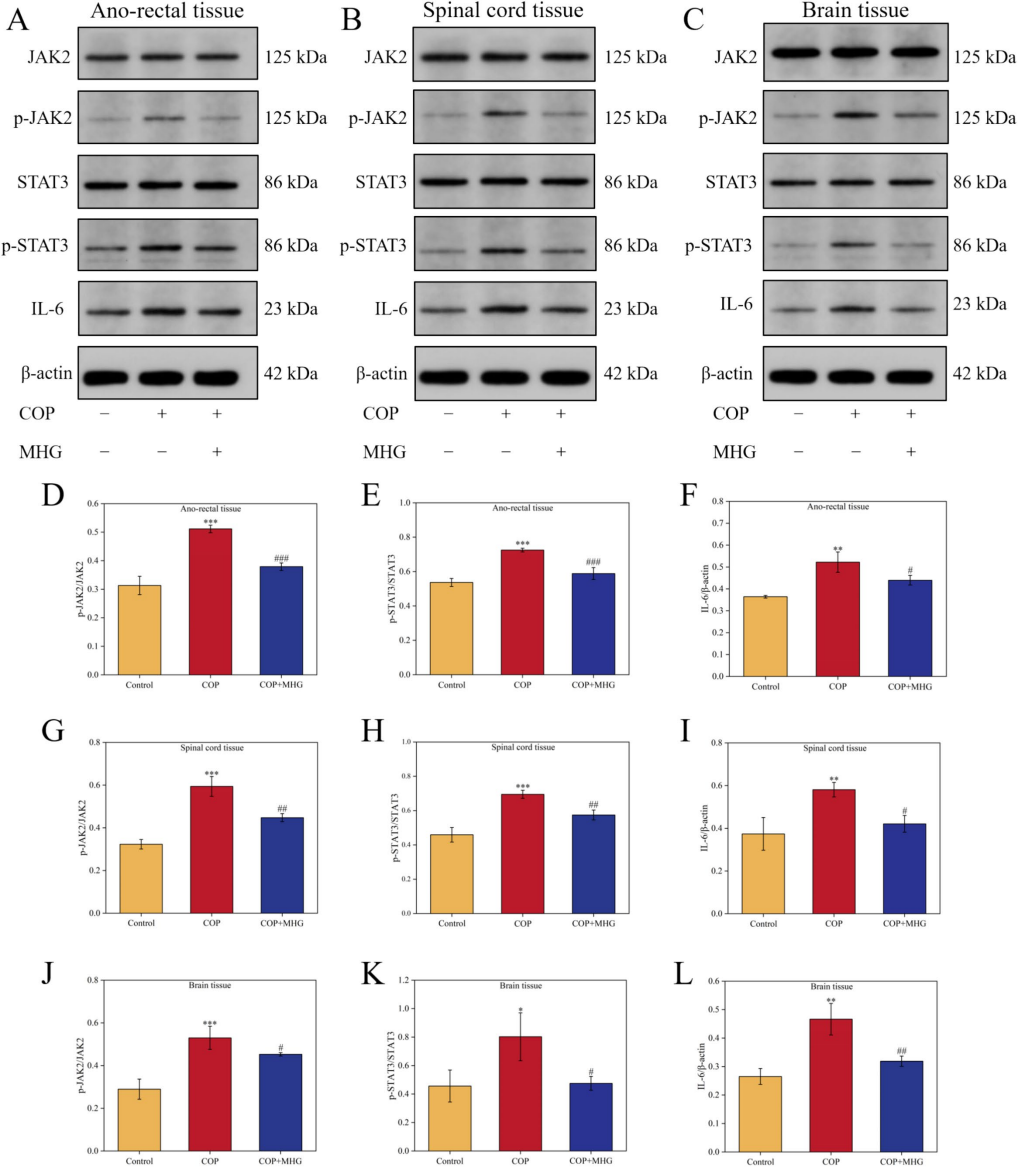
The effect of MHG on the expression of JAK2/STAT3 signaling pathway related proteins in rectal epidermis-spinal brain axis. The representative bands of JAK2, p-JAK2, STAT3, p-STAT3, and IL-6 were determined by Western blotting in ano-rectal tissue (A), spinal cord tissue (B) and brain tissue (C). Protein relative expression level of p-JAK2/JAK2 (D), p-STAT3/STAT3 (E), and IL-6 (F) in ano-rectal tissue. Protein relative expression level of p-JAK2/JAK2 (G), p-STAT3/STAT3 (H), and IL-6 (I) in spinal cord tissue. Protein relative expression level of p-JAK2/JAK2 (J), p-STAT3/STAT3 (K), and IL-6 (L) in brain tissue (*n* = 3). Data are presented as the mean ± SD, Compared with the control group; ^*^p<0.05, ^**^p < 0.01, ^***^p < 0.001, Compared with the COP group, *^#^p* < 0.05, *^##^p* < 0.01*, ^###^p* < 0.001. Uncropped raw data can be found in Supplementary Figure S4.

Network pharmacological analysis results indicate that the JAK/STAT signaling pathway may be a key pathway for MHG to alleviate PA. Therefore, we further used Western Blotting to detect the relative expression levels of JAK2, p-JAK2, STAT3, p-STAT3, and IL-6 proteins in the rectum, spinal cord, and brain tissues of hemorrhoid rats, as shown in Figures 5A–C. From Figures 5D–F, in the rectal tissues of rats, the relative expression levels of p-JAK2/JAK2, p-STAT3/STAT3, and IL-6 protein in the COP group were notably elevated when comparing with Control group (*p* < 0.01, *p* < 0.001). Treatment with MHG remarkably decreased the relative expression levels of p-JAK2/JAK2, p-STAT3/STAT3, and IL-6 protein when comparing with COP group (*p* < 0.05, *p* < 0.001). Interestingly, similar results were observed in the spinal cord and brain tissues of hemorrhoid rats (Figures 5G–L), indicating that MHG can inhibit the activation of the JAK2/STAT3 signaling pathway in the rectal epidermis-spinal cord-brain axis of hemorrhoid rats.

## 4 Discussion

At the outset of this study, we employed various methods to establish a rat model of PA, including the application of croton oil to the epidermis to induce itching responses. However, we observed that this method did not produce significant pruritic behaviors. After reviewing relevant literature, we shifted to a chloroquine-induced mouse itching model, which successfully elicited pronounced itching behaviors. This model allowed for a clearer assessment of the anti-itching effects of the drug. Therefore, two animal models were selected in this study to explore the therapeutic efficacy and mechanism of MHG in relieving PA.

Hemorrhoids, as the most common anal and rectal disease clinically, are increasingly prevalent across all age groups, with intense pain and itching being the main reasons for reducing patients’ quality of life (28, 29). It has been reported that croton oil can disrupt the mucosa of rat rectal tissues and induce anal swelling, which is consistent with the pathological features of clinical hemorrhoids (30). Injection of chloroquine into the neck and back skin of mice can induce significant scratching behavior, resulting in a non-histamine-dependent itching response, and is commonly used to observe animal itching behavior (31). After MHG treatment, we observed significant improvements in hemorrhoidal swelling and a reduction in scratching behavior in mice, indicating efficacy of MHG in alleviating PA. Additionally, MHG significantly improved chloroquine-induced skin epidermal thickening and inflammatory cell infiltration in mice. It also alleviated COP-induced rectal tissue swelling, bleeding, and inflammatory response in rats and restored intestinal villus structure and intestinal barrier.

Subsequently, we utilized network pharmacology analysis to investigate the potential mechanisms of MHG in alleviating PA. Among the 20 identified compounds, Trigalloyl hexose, Tetragalloyl hexose, and Hamamelitannin, as tannin derivatives, exhibit significant antibacterial properties, which help improve symptoms of bacterial infection around the anus (32). Rutin, Kaempferol 3-O-glucoside, Kaempferol, and Naringenin belong to the flavonoid class of compounds. Due to their excellent anti-inflammatory effects, they effectively maintain the integrity of the intestinal barrier and suppress inflammation in the intestines (33, 34). Protocatechuic acid, Ethyl gallate, Ferulic acid, Theogallin, Chlorogenic acid, and Gallic acid, which are phenolic acid substances present in MHG, possess various pharmacological activities, including antioxidant, anti-inflammatory, platelet aggregation promotion, and acceleration of skin tissue repair processes (35, 36). Additionally, terpenoid components in MHG, such as (+)-Fenchol, (+/−)-Camphor, (+)-Borneol, (+)-borneol, and (−)-Borneol, not only exhibit anti-inflammatory, antioxidant, anti-apoptotic, and anticoagulant activities but also improve energy metabolism and enhance transdermal drug absorption by increasing skin permeability, thereby enhancing therapeutic effects (37). In summary, the active ingredients in MHG exert their efficacy against PA through multi-pathway and multi-target mechanisms, providing scientific basis for clinical treatment.

To further explore the mechanism of MHG, we analyzed the compound-target-disease network and identified 60 key genes to construct a protein–protein interaction network. Key genes such as BCL2, STAT3, and JUN were found to be closely associated with the pathogenesis of PA. BCL2 is known to regulate cell apoptosis, and studies have indicated that its overexpression in lymphocytes is linked to the development and progression of skin itching-related diseases such as urticaria and specific dermatitis (38, 39). STAT3 is a crucial regulatory factor in itch pathogenesis, as its activation can promote the release of inflammatory mediators and alter neurotransmitter levels, contributing to the sensation and worsening of itching (40). JUN, a member of the AP-1 transcription factor family, plays a role in cell proliferation, differentiation, and apoptosis. The activation of JUN in epidermal cells may lead to increased release of inflammatory mediators, which can exacerbate skin itching (41). According to GO and KEGG pathway analysis, these targets are associated with signal transduction and inflammation, including the PI3K/Akt signaling pathway, JAK/STAT signaling pathway, and Ras signaling pathway. Consistent with previous studies, the JAK2/STAT3 signaling pathway is known to contribute to the development of itching-related skin diseases through various mechanisms, such as regulating inflammation, affecting keratin formation, and influencing immune responses, making it an important regulatory factor in the occurrence and progression of itching-related skin diseases (42–44). Therefore, regulation of the JAK2/STAT3 signaling pathway may offer potential therapeutic benefits for treating and alleviating itching-related diseases, and this hypothesis will be further validated in subsequent experiments.

The Skin-Brain Axis refers to the complex network of bidirectional communication between the skin and the brain through the nervous system, endocrine system, and other signaling molecules (45). This concept emphasizes that the skin is not only the body’s external protective layer but also communicates with the central nervous system (CNS) by releasing various signaling molecules such as neurotransmitters, cytokines, and hormones, playing an important role in the pathogenesis and regulation of skin diseases, mental disorders, and immune-related diseases (46, 47). The rectal epidermis-spinal cord-brain axis, as an important component of the Skin-Brain Axis, mainly processes and responds to sensory information related to the rectum, such as itching, pain, and temperature. Using a rat hemorrhoid model, we examined the correlation of key proteins in TRP channels (an important itching signaling pathway) expressed in the rectum epidermis-spinal cord-brain axis and confirmed the mechanism of MHG in improving PA is related to the regulation of TRP channels. We found that the protein expression levels of TRPV1 and TRPA1 in the rectum of hemorrhoid model rats were significantly increased, while the protein expression level of TRPM8 was significantly decreased. Consistent with literature reports, TRPV1 and TRPA1 are activated by stimuli such as inflammation and chemicals, participating in itch signal transmission, while TRPM8 is a cold stimulus receptor channel that is activated by cooling and alleviates itching (48–50). MHG treatment significantly downregulated the protein expression of TRPV1 and TRPA1 while upregulating the protein expression level of TRPM8, indicating that MHG can alleviate PA by regulating the expression of TRP channel-related proteins in the rectum. Additionally, the Borneolum in MHG can also improve skin permeability and enhance drug absorption by activating TRPM8 (51).

Subsequently, to explore the transmission of itching signals in the peripheral spinal cord, we further examined the expression of proteins related to the GRP/GRPR pathway in the spinal cord tissue of hemorrhoid model rats. GRP/GRPR is a specific signaling pathway for the transmission of itching signals in the spinal cord. When GRPR-expressing neurons in the spinal cord are inhibited, mice do not exhibit any scratching behavior (52, 53). Similarly, we found that the protein expression of c-Fos and GRPR in the spinal cord of hemorrhoid model rats was significantly increased, while MHG treatment significantly downregulated the protein expression of c-Fos and GRPR. This indicates that MHG inhibits the activation of GRPR-positive neurons and can alleviate PA by regulating the spinal cord GRP/GRPR pathway. Finally, we detected the protein expression of 5-HT1a in the brain of hemorrhoid model rats to confirm the involvement of the rectal-spinal cord-brain axis in the alleviation of PA by MHG. 5-HT is considered a potent factor in inducing itching, and 5-HT1a receptor is a G protein-coupled receptor that plays an important role in itching signals regulated by 5-HT. (54) Additionally, Zhao et al. (8) demonstrated that activation of 5-HT1a promotes GRP/GRPR signal transduction and increases the persistence of itching output. In this study, we observed an increase in 5-HT1a expression in the brain tissue of hemorrhoid model rats, and MHG intervention significantly downregulated the expression of 5-HT1a, indicating an inhibitory effect of MHG on the activation of 5-HT1a. In summary, these findings suggest that the rectal epidermis-spinal cord-brain axis plays an important role in the transmission of PA signals, and MHG can alleviate PA symptoms by regulating this axis.

To validate the results of the network pharmacology analysis, we further examined the effects of MHG on the JAK2/STAT3 signaling pathway within the rectal epidermis-spinal cord-brain axis. The JAK2/STAT3 pathway is a crucial mechanism for intracellular signal transduction and is closely linked to various itching-related diseases, such as psoriasis, specific dermatitis, and nodular prurigo (55–57). Previous studies have shown that disrupting STAT3 in astrocytes significantly reduces GRP-induced itching, and clinical use of JAK2 inhibitors has been found to improve itching symptoms in patients with specific dermatitis (58, 59). In our study, we observed that phosphorylation levels of JAK2 and STAT3 were elevated in the rectum, spinal cord, and brain tissues of hemorrhoid model rats, along with increased expression levels of IL-6. This indicates activation of the JAK2/STAT3 signaling pathway within the rectal epidermis-spinal cord-brain axis. Treatment with MHG resulted in the inhibition of JAK2 and STAT3 phosphorylation, a reduction in IL-6 expression, and suppression of the JAK2/STAT3 signaling pathway activation. These findings suggest that MHG may alleviate itching by regulating the JAK2/STAT3 signaling pathway, which in turn inhibits the transmission of itching signals within the rectal epidermis-spinal cord-brain axis. This not only supports the network pharmacology analysis results but also underscores the potential of MHG as an effective treatment for itching-related symptoms.

However, there are some limitations in this study. Firstly, we only investigated the therapeutic effects of MHG in an acute hemorrhoid/itching model, and the short observation period may present a limitation. In the future, we plan to gradually extend the treatment period to 3, 5, or even 7 days to further assess its long-term efficacy and potential adverse effects in PA. This will allow for a more comprehensive validation of its therapeutic benefits and safety. Secondly, considering ethical concerns, the sample size of rats in this study was relatively small. Nevertheless, we adhered to a randomized grouping design, with 3 rats per group, ensuring the statistical reliability of the results. In future studies, we will increase the sample size to further explore the therapeutic mechanisms of MHG and enhance the depth and breadth of the current findings.

In conclusion, our study demonstrates that MHG effectively alleviates PA, likely through the inhibition of JAK2/STAT3 signaling pathway activation. This mechanism appears to suppress the transmission of itching signals along the rectal epidermis-spinal cord-brain axis, resulting in an anti-pruritic effect. However, given the complex nature of itching pathogenesis, further research employing gene-editing techniques such as CRISPR/Cas9, RNA interference (RNAi), and TALEN is needed to validate and expand upon the findings of this study.

## Data Availability

The original contributions presented in the study are included in the article/Supplementary material, further inquiries can be directed to the corresponding author.

## Footnotes

^1^ https://pubchem.ncbi.nlm.nih.gov/

^2^ http://swisstargetprediction.ch/

^3^ https://www.genecards.org/

^4^ https://www.omim.org/

^5^ https://www.disgenet.org/

^6^ https://bioinfogp.cnb.csic.es/tools/venny/

^7^ https://string-db.org/

^8^ https://david.ncifcrf.gov/

^9^ http://www.bioinformatics.com.cn/
