# Supplementary material for: Mechanism of medical hemorrhoid gel in relieving pruritus ani via inhibiting the activation of JAK2/STAT3 pathway
Source: Front Med (Lausanne). 2024 Nov 13;11:1487531. doi: 10.3389/fmed.2024.1487531 (PMC11600105; doi:10.3389/fmed.2024.1487531)
Supplement: Supplementary file 1 [file Data_Sheet_1.pdf]

Supplemental Information for: Mechanism of medical hemorrhoid gel in relieving hemorrhoidal pruritus via inhibiting the activation of JAK2/STAT3 pathway

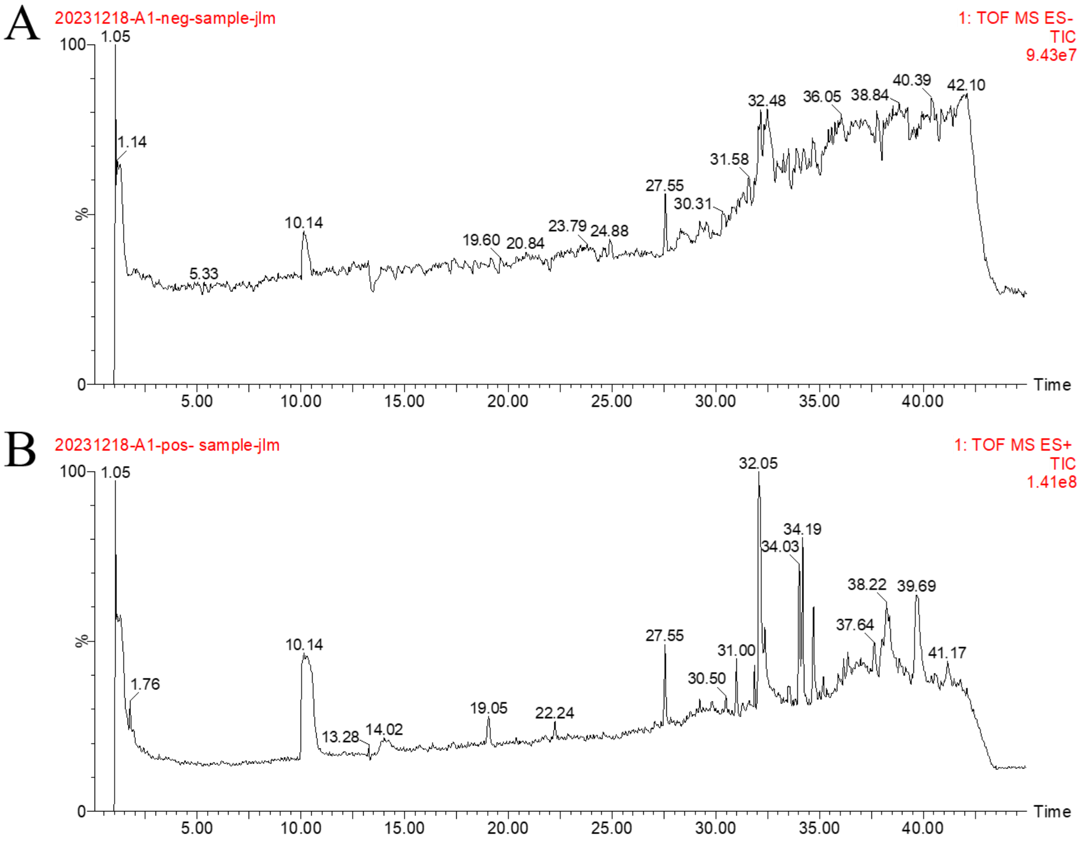

Figure S1. Total ion flow chromatograms of MHG analyzed by UPLC-QTOF-MS/MS in negative (A) and positive (B) ion modes.

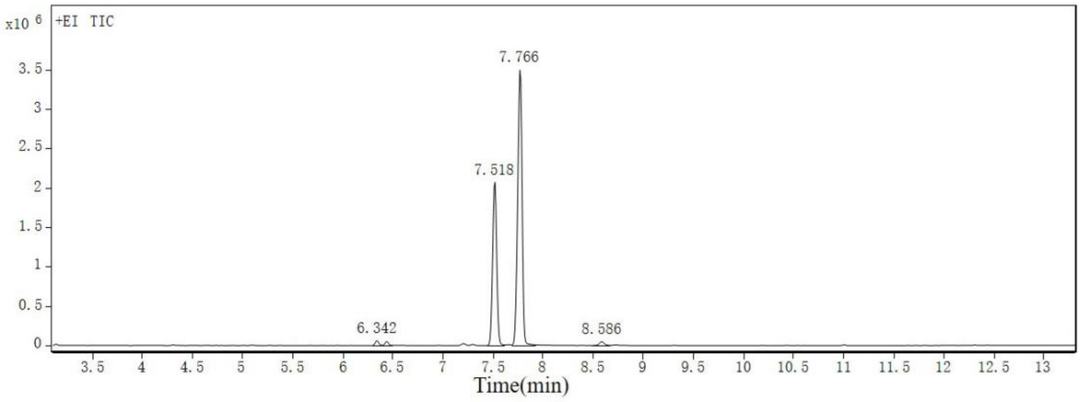

Figure S2. Total ion chromatogram of f MHG analyzed by GC-MS/MS

Table S1. The information of 20 compounds identified of MHG

Compound	t_R (min)	Polarity	Formula	Molecular weight	Class	Degree
Trigalloylhexose	29.22	$[M-H]^-$	$C_{27}H_{24}O_{18}$	636.46	Tannins	5
Tetragalloylhexose	38.22	$[M+H]^+$	$C_{34}H_{28}O_{22}$	788.58	Tannins	5
Hamamelitannin	20.38	$[M+H]^+$	$C_{20}H_{20}O_{14}$	478.41	Tannins	7
Protocatechuic acid	1.76	$[M+H]^+$	$C_7H_6O_4$	154.11	Phenolic acids	15
Ethyl gallate	10.14	$[M+H]^+$	$C_9H_{10}O_5$	198.18	Phenolic acids	17
Ferulic acid	12.07	$[M+H]^+$	$C_{10}H_{10}O_4$	194.19	Phenolic acids	35
Theogallin	35.58	$[M-H]^-$	$C_{14}H_{16}O_{10}$	344.27	Phenolic acids	7
Chlorogenic acid	37.64	$[M+H]^+$	$C_{16}H_{18}O_9$	354.32	Phenolic acids	14
Gallic acid	41.32	$[M-H]^-$	$C_7H_6O_5$	170.11	Phenolic acids	15
(+)-Fenchol	6.441	$[M+H]^+$	$C_{10}H_{18}O$	154.14	Natural terpenoids	20
Camphor	7.203	$[M+H]^+$	$C_{10}H_{16}O$	152.12	Natural terpenoids	14
(+)-Borneol	7.518	$[M+H]^+$	$C_{10}H_{18}O$	154.14	Natural terpenoids	20
(-)-Borneol	7.775	$[M+H]^+$	$C_{10}H_{18}O$	154.14	Natural terpenoids	20
Rutin	29.22	$[M+H]^+$	$C_{27}H_{30}O_{16}$	610.53	Flavonoids	7
Gallocatechin	31.08	$[M-H]^-$	$C_{15}H_{14}O_7$	306.26	Flavonoids	__
Kaempferol 3-O-glucoside	33.26	$[M-H]^-$	$C_{21}H_{20}O_{11}$	448.37	Flavonoids	14
Naringenin	37.76	$[M-H]^-$	$C_{15}H_{12}O_5$	272.24	Flavonoids	57
Kaempferol	39.25	$[M-H]^-$	$C_{15}H_{10}O_6$	286.23	Flavonoids	62
2,5-Dimethyl-2,4-hexadiene	8.586	$[M+H]^+$	C_8H_{14}	110.11	Olefin derivatives	3
4-Ethyl-3-oxabicyclo[4.4.0]decane	6.342	$[M+H]^+$	$C_{11}H_{20}O$	168.15	Epoxy	9

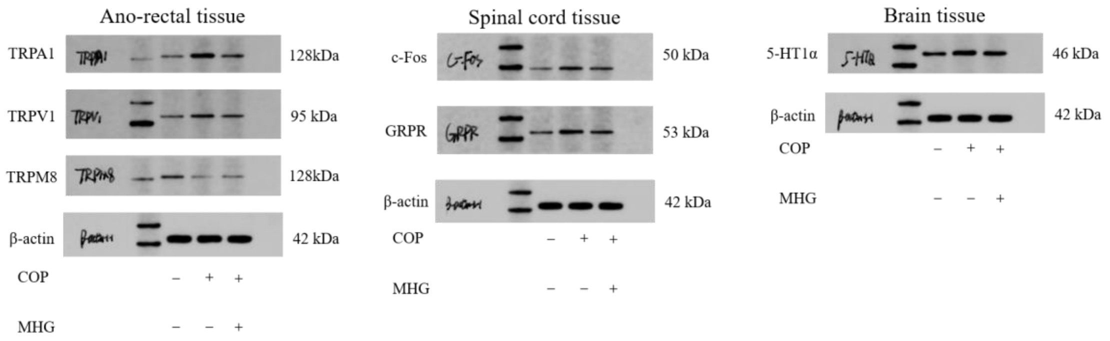

Figure S3. Rectal-spinal-brain axis related proteins-WB-rawdata.

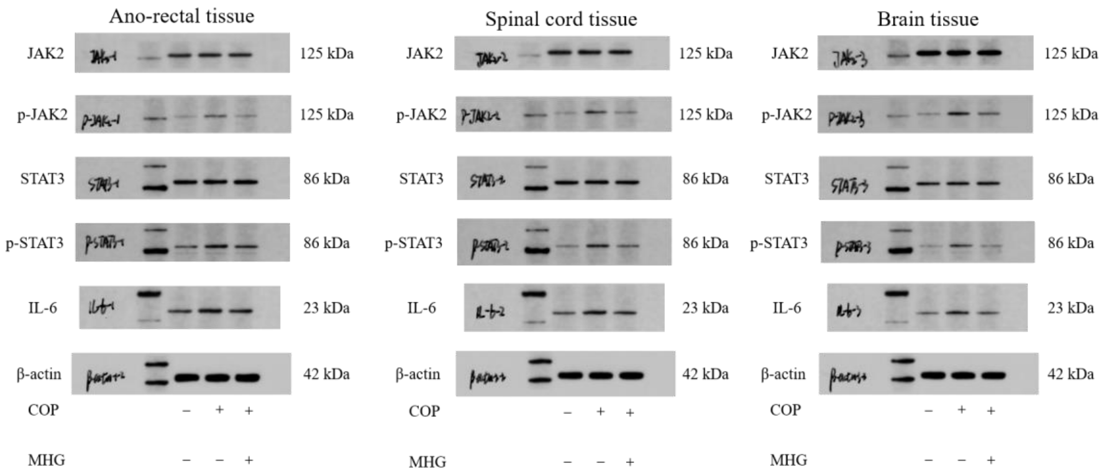

Figure S4. JAK2/STAT3 signal pathway related proteins-WB-rawdata.
